# Dietary Strategies for Obesity

**DOI:** 10.3390/nu15194275

**Published:** 2023-10-07

**Authors:** Jacqueline L. Walker, Robyn Littlewood

**Affiliations:** 1 Health and Wellbeing Queensland, Queensland Government, Brisbane, QLD 4064, Australia; 2 School of Human Movement and Nutrition Sciences, The University of Queensland, St Lucia, QLD 4072, Australia

Overweight and obesity remains an important health focus internationally, due to the strong link to many noncommunicable diseases, such as cardiovascular disease, non-alcoholic fatty liver disease, diabetes mellitus and mental health conditions [1]. The World Health Organization 2030 Agenda for Sustainable Development highlights noncommunicable diseases as a major challenge for sustainable development, with goal 3.4 stating “By 2030, reduce by one third premature mortality from non-communicable diseases through prevention and treatment and promote mental health and well-being” [2]. Statistics concerning overweight and obesity continue to either rise or remain stable in most countries across the globe, however, what has not been able to be achieved is a decrease in prevalence [3]. Worldwide obesity has nearly tripled since 1975 [1], and recent global estimates indicate that over 51% of the world’s population will be living with overweight (27%) or obesity (24%) by 2035 [3]. The economic impacts of this are immense, estimated to be US$ 4.32 trillion or 2.9% of total global GDP by 2035 [3].

As recommended by numerous international organisations committed to the prevention and management of overweight and obesity, multiple solutions at varying levels of government and society are required to accelerate action on obesity [3]. The ROOTS Framework, as developed by the World Obesity Federation in consultation with its members in 2020, represents an ideal approach to addressing obesity (Figure 1) [4]. The Declaration that was reached at the 2020 Global Obesity Forum details recommendations under each of these five pillars that can be applied across countries [4]. While action under each pillar is paramount, so is the research that is required to evidence, support, and explain these actions and their outcomes. Research in the area of overweight and obesity can often be seen as ‘less attractive’ when compared to other health conditions due to the complexity of the issue. It is these complexities, however, that need to be unravelled, understood, and then accounted for in order to successfully drive innovative solutions.

This Special Issue of Nutrients focuses on ‘Dietary Strategies for Obesity’, which can encompass various diet and food-related approaches that could be applied under four of the five pillars of the ROOTS Framework (the exception being the recognition of obesity). It is important that the scope of research work remains wider than just that of treatment approaches, to effectively capture strategies that work across the human lifespan, at multiple system levels, and are contextualised to the community and/or country.

Australia is a contemporary example of a developed nation which is actively addressing overweight and obesity across all pillars of the ROOTS Framework. From a national perspective, the Australian federal government released the National Obesity Strategy in 2022, which details a 10-year plan to enable Australian to eat well and be active. Current Australian statistics are concerning, with two in three adults [5] and one in four children [6] living with overweight or obesity, and to cover the cost of this, each Australian pays an additional $678 in taxes per year [7]. All state and territory governments have individual jurisdiction to apply the National Obesity Strategy in varied ways, and many are currently working towards contextualising the strategy and taking action. For Queensland, the second largest state in Australia, a more unique challenge presents itself due to the relatively decentralised population compared to other states [8], where 22% of adults living in outer regional areas and 50% of those in remote areas are living with obesity [9]. These health disparities must be considered when designing strategies and solutions across all pillars of the ROOTS Framework [4], particularly when understanding factors that can influence dietary strategies such as food availability and cost, health service accessibility, and local support systems.

The studies featured in this Special Issue are critical in furthering our understanding of key dietary strategies that can be used across populations, systems, and societies, according to the ROOTS Framework, to address overweight and obesity. Findings have the potential to assist in large scale areas such as the policy environment, the development of evidence-based guidelines, and the application of funding in political settings, but most importantly, to improve the health and wellbeing of people across their lifetime.

## Figures and Tables

**Figure 1:**
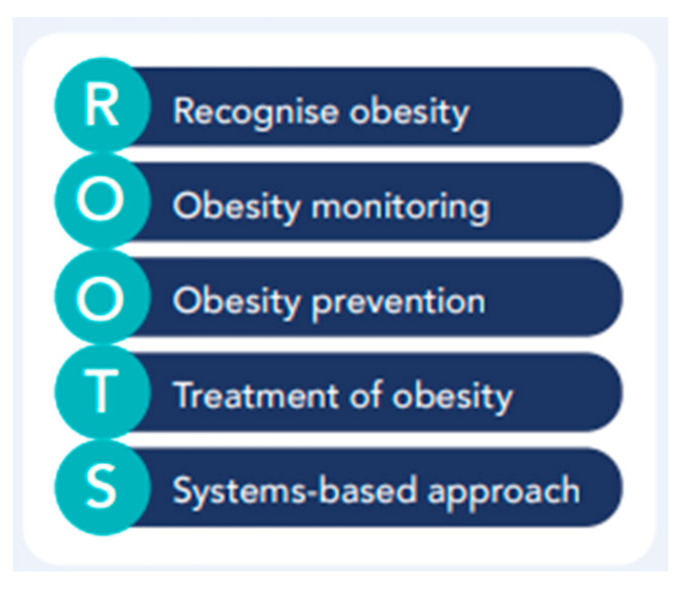
The five pillars of the ROOTS Framework; an integrated, equitable, comprehensive and person-centred approach to addressing obesity [4]. Reproduced with permission from the World Obesity Federation, 2023.
